# Well-leg compartment syndrome after orthopedic surgery in the hemi-lithotomy position: a three-case series and literature review

**DOI:** 10.1093/jscr/rjag485

**Published:** 2026-06-30

**Authors:** Radu Dumitru Antoniu, Filippo-Franco Schiapparelli, Philippe Vial, Markus Simon Hanke

**Affiliations:** Sonnenhof Clinic of Orthopaedics, Medical Center Biel, Vogelsang 84, 2502 Biel, Switzerland; Medical Center Vigimed, Rue du Léman 18B, 1920 Martigny, Switzerland; Department of Orthopaedics, HFR Fribourg Cantonal Hospital, Chemin des Pensionnats 2/6, 1752 Villars-sur-Glâne, Switzerland; Sonnenhof Clinic of Orthopaedics, Medical Center Biel, Vogelsang 84, 2502 Biel, Switzerland

**Keywords:** well-leg compartment syndrome, WLCS, hemi-lithotomy, fasciotomy

## Abstract

Well-leg compartment syndrome (WLCS) is an acute compartment syndrome developing in an uninjured limb, classically associated with lithotomy or hemi-lithotomy positioning. We report three cases of WLCS following femoral fixation performed on a traction table with the contralateral limb positioned in hemi-lithotomy and review previously reported orthopedic and trauma-related cases. Diagnostic, treatment, and prevention recommendations are derived from the most recent available literature. Most reported orthopedic cases occur after femoral intramedullary nailing. Reported risk factors include prolonged continuous elevation (≥4 h), increased body mass-index, head-down tilt, hypovolemia, forced ankle dorsiflexion, calf-supported positioning, intraoperative hypotension, and increased external pressure. WLCS appears to be largely preventable. Diagnosis is primarily clinical, and fasciotomy should not be delayed. When indicated, two-incision, four-compartment decompression remains the recommended approach, whereas selective fasciotomy is discouraged.

## Introduction

**Figure 1:**
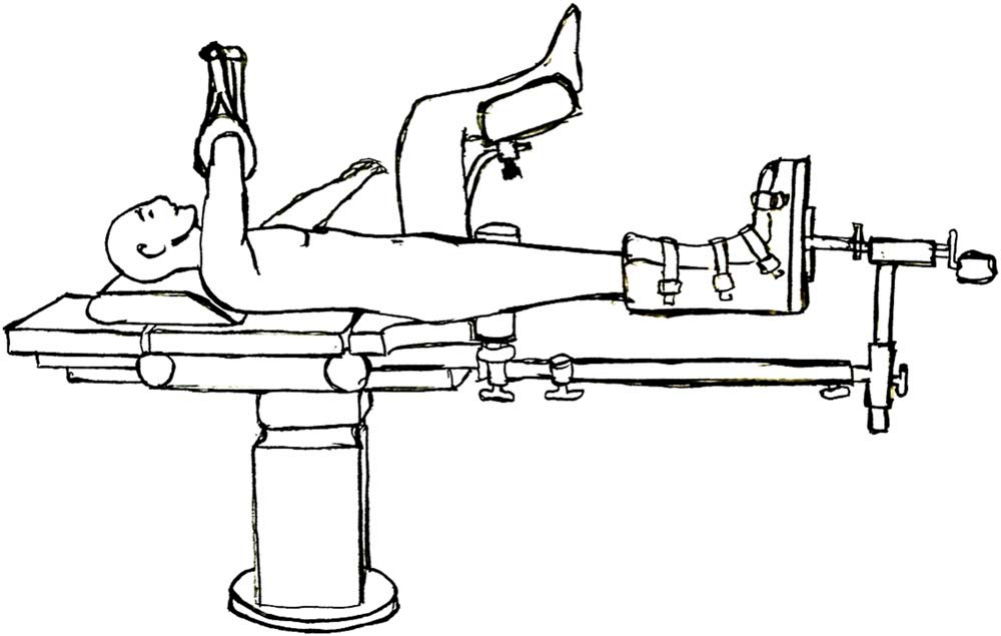
Body positioning on the traction table in the hemi-lithotomy position (R.-D. Antoniu).

Well-leg compartment syndrome (WLCS) refers to an acute compartment syndrome developing in an uninjured limb after surgery. This condition was recognized across multiple surgical specialties [1]. In orthopedic trauma surgery, WLCS is most frequently reported after femoral fixation performed on a traction table with the contralateral ‘well’ leg positioned in hemi-lithotomy (Fig. 1). Although this setup facilitates fluoroscopic imaging, it may reduce perfusion pressure in the elevated limb, increase external compression from the limb support, and raise intracompartmental pressures [2–4]. Although uncommon, WLCS is likely under-recognized and delayed diagnosis may result in irreversible neuromuscular deficits and systemic complications [3–5]. In this article, we report three cases of WLCS that occurred after femoral fixation on a traction table in two institutions. We also review previously reported orthopedic and trauma-related WLCS cases and discuss current concepts regarding pathophysiology, diagnosis, treatment, and prevention.

## Case series

### Case 1

**Figure 2:**
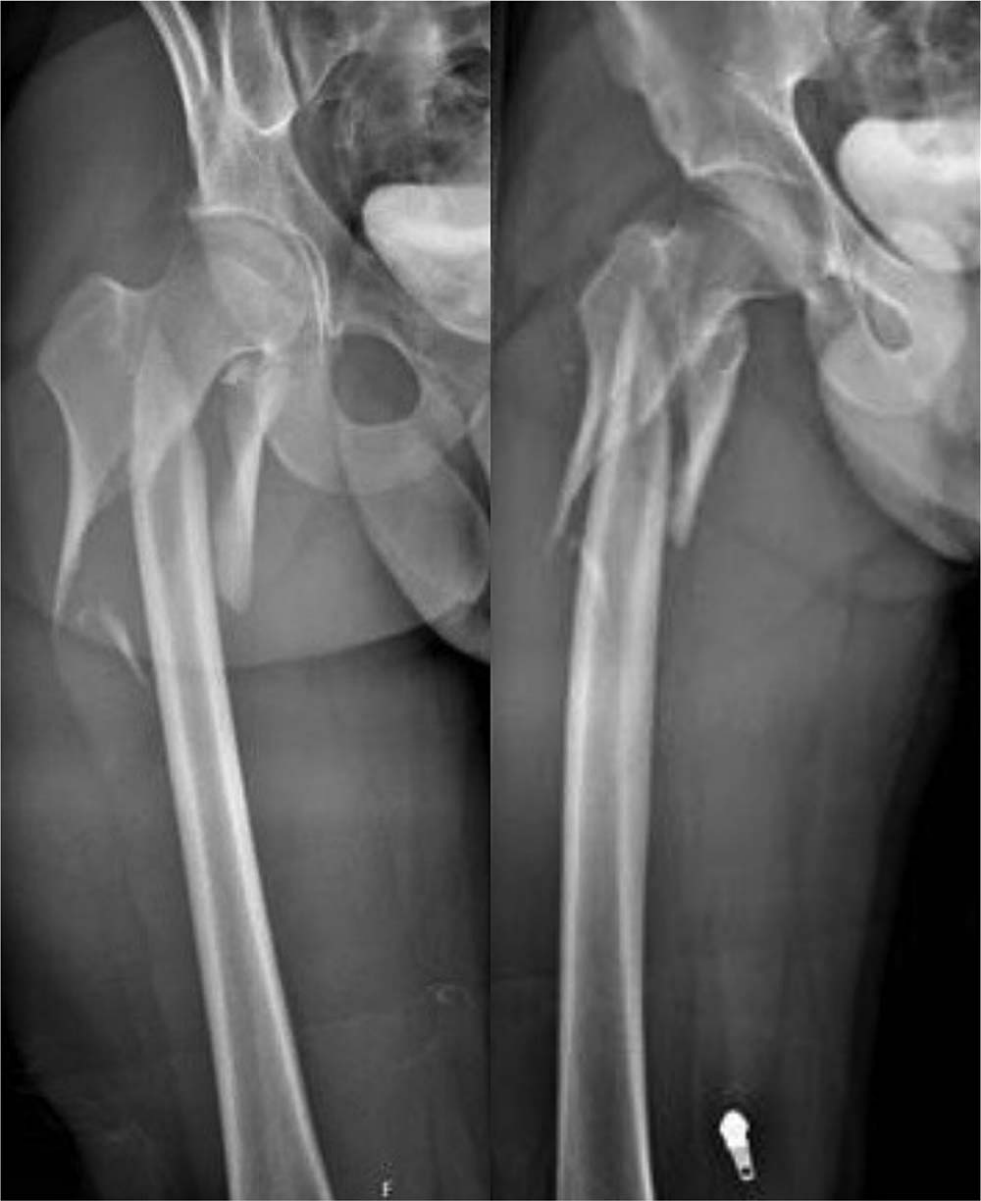
Pre-operative anteroposterior (left) and lateral (right) radiographs of the injured femur.

**Figure 3:**
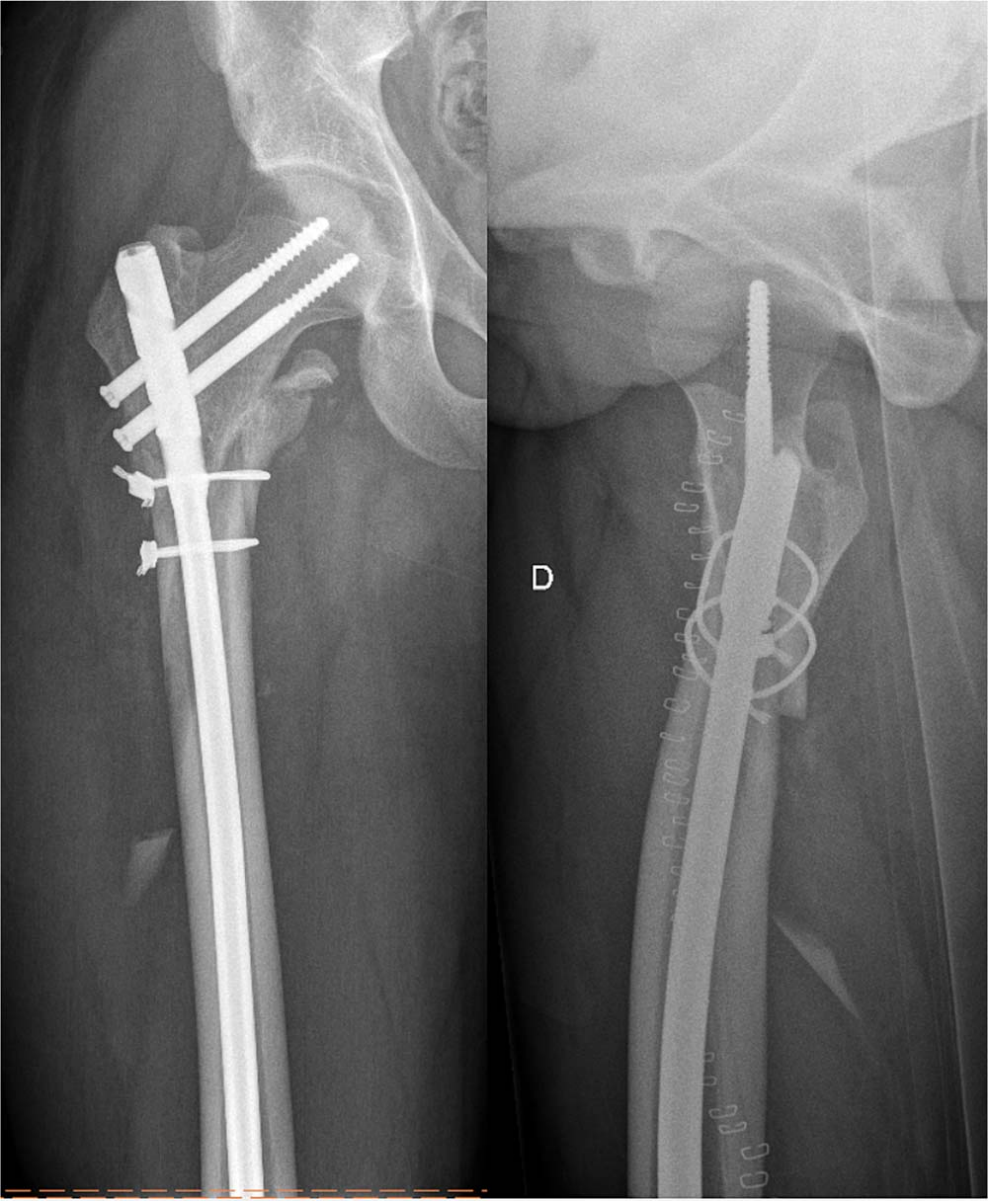
Post-operative anteroposterior (left) and lateral (right) radiographs of the injured femur.

A 20-year-old male, with a body mass index (BMI) of 23.5 kg/m^2^, sustained a reverse multifragmentary intertrochanteric fracture (AO/OTA 31-A3.3) (Fig. 2), with ipsilateral fractures of the second to fourth metatarsal heads following a motor-vehicle accident. Preoperative lactate was 1.6 mmol/L. Open reduction with two Dall-Miles cerclage cables and fixation using a long femoral nail (diameter 10 mm, length 400 mm) were performed under general anesthesia with the patient positioned on a traction table in hemi-lithotomy position, with the contralateral leg supported under the calf (Fig. 3). Operative time was 7 h with estimated blood loss of 600 mL. No intra-operative hypotension occurred. Eight hours postoperatively, the patient developed severe pain in the contralateral calf. WLCS was diagnosed clinically without compartment pressure measurements. Emergent two-incision, four-compartment fasciotomy was performed. The medial wound was closed primarily; the lateral wound subsequently became infected and required repeat debridement followed by split-thickness skin grafting. At 6-month follow-up, the patient had returned to full function.

### Case 2

A 15-year-old male, BMI 21.1 kg/m^2^, sustained a proximal femoral shaft wedge fracture (AO/OTA 32-B3a) after a ski accident. Preoperative lactate was 2.1 mmol/L. Open reduction and lateral-entry femoral nailing (diameter 8.2 mm, length 300 mm) were performed under general anesthesia in hemi-lithotomy with calf support. Operative time was 4 h 39 minutes and estimated blood loss was 300 mL. No intraoperative hypotension occurred. Four hours postoperatively, the patient developed acute pain in the contralateral calf. WLCS was suspected and compartment pressure measurements showed 70 mmHg in the lateral compartment and 30 mmHg in the anterior compartment. Emergent two-incision, four-compartment fasciotomy was performed, with primary closure of the wounds. At 6-month follow-up, the patient was asymptomatic.

### Case 3

A 39-year-old male, BMI 30.8 kg/m^2^, sustained a transverse mid-shaft femoral fracture (AO/OTA 32-A3). Closed reduction and intramedullary fixation with a T2 Alpha nail (diameter 13 mm, length 440 mm) were performed on a traction table with the patient in hemi-lithotomy position and the contralateral leg supported under the calf. Operative time was 4 h 7 min. During the first 90 min of surgery, sustained hypotension occurred, with arterial pressures as low as 85/40 mmHg, requiring norepinephrine administration (40 μg). In the post-anesthesia care unit, the patient developed severe pain in the contralateral leg that was refractory to intravenous fentanyl. Tense swelling of the lateral and posterior compartments was noted. WLCS was suspected and confirmed by intracompartmental pressure measurements of 32 mmHg in the anterior compartment, 75 mmHg in the lateral compartment, 46 mmHg in the superficial posterior compartment, and 35 mmHg in the deep posterior compartment. Emergent two-incision, four-compartment fasciotomy was performed within 1 h of diagnosis. The lateral wound was closed on postoperative day 2, whereas the medial wound required negative-pressure wound therapy and was closed on postoperative day 24. At 6 months, no residual sensorimotor deficit was present.

## Discussion

**Figure 4:**
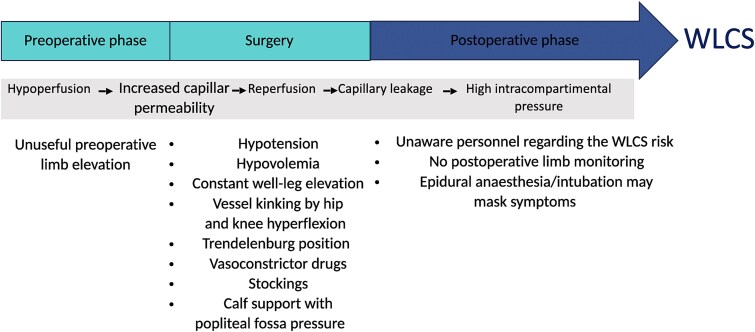
Pathophysiology of WLCS and modifiable perioperative risk factors.

In WLCS, tissue hypoperfusion arises from a reduction in perfusion pressure due to elevation of the limb in the hemi-lithotomy position, a situation that may be further aggravated by a Trendelenburg tilt. The resulting reduction in tissue oxygen delivery forces cells to rely on anaerobic metabolism, leading to accumulation of lactate and other intermediate metabolites. Ongoing ischaemia disrupts capillary endothelial integrity, permitting extravasation of fluid and plasma proteins into the interstitial space, resulting in interstitial oedema and progressive elevation of intracompartmental pressure (Fig. 4) [6].

Calf and knee supports impose higher local pressures on the posterior compartments than heel- or ankle-based supports, and prolonged contact further increases intramuscular pressure over time. Tan *et al*. demonstrated that simply placing the non-operative limb into a calf-supporting holder increased compartment pressures from ~9 mmHg to ~27 mmHg [7]. Meyer *et al*. showed that changing from a calf-supported to a heel-supported hemi-lithotomy position reduced pressures in all compartments by ˃10 mmHg, indicating that direct calf compression is a major contributor [2]. Circumferential compression from stockings may also contribute to the reduced perfusion of the limb.

Limb elevation reduces arterial pressure at the ankle. Meyer *et al*. reported that moving the leg from a supine position into lithotomy increased compartment pressure from 9 mmHg to 27 mmHg [2]. In addition, perfusion pressure within the leg decreases by roughly 0.78 mmHg for each centimeter the limb is elevated above the level of the right atrium, corresponding to a reduction of ~24 mmHg at 30.5 cm elevation [8].

Compartment pressures increase progressively with continued elevation. Clinical series suggest that the risk of WLCS increases substantially when the surgery time approaches or exceeds 4 h. Tan *et al*. also observed a continuous rise in the compartment pressure of the well-leg of 1.1 mmHg per hour during the procedure [7].

Forced ankle dorsiflexion increases compartment pressures, whereas neutral or slightly plantarflexed ankle position is preferred [9]. Flexion at the hip and knee may also contribute by reducing arterial inflow through kinking of the femoral and popliteal vessels. Increased body weight has also been associated with higher intracompartmental pressures in the hemi-lithotomy position [7]. Patients with established peripheral arterial disease of the lower limbs may be particularly susceptible to additional reductions in tissue perfusion when placed in the lithotomy position, especially if combined with a head-down tilt, thereby increasing their risk of WLCS. With prolonged ischaemia, endothelial injury and oedema develop; reperfusion then amplifies swelling in closed fascial spaces, increasing pressures and risking irreversible muscle and nerve injury.

Intraoperative hypotension, hypovolemia, vasoconstrictor use, and Trendelenburg positioning may all further reduce perfusion to the elevated limb and should be minimized where possible. While restrictive fluid strategies may offer benefits regarding potential postoperative complications or infection risk [10], excessive fluid restriction may compromise tissue perfusion and aggravate hypotension; consequently, strict perioperative fluid restriction should be avoided in patients undergoing prolonged surgery with an elevated limb.

Diagnosis is primarily clinical and must not be delayed. Severe pain out of proportion, pain on passive stretch, tense compartments, and, in evolving cases, paraesthesia or weakness should prompt immediate evaluation. In awake patients, serial clinical examination remains decisive. When the clinical examination is unreliable or equivocal, compartment pressure measurements may support decision-making. A differential pressure (ΔP = diastolic blood pressure – intracompartmental pressure) of 30 mmHg or less in any compartment is consistent with acute compartment syndrome and supports urgent fasciotomy. WLCS should be treated as a surgical emergency, with fasciotomy undertaken ideally within 1 h of diagnosis. A fasciotomy performed ˃12 h after symptom onset is associated with a higher risk of permanent deficits, whereas decompression within 6 h of diagnosis offers the best chance of full functional recovery. Treatment principles are identical to those of acute compartment syndrome; the recommended standard is an urgent two-incision, four-compartment fasciotomy. Selective fasciotomy is discouraged because of the risk of unrecognized multi-compartment involvement. A planned second-look procedure at 48–72 h to reassess muscle viability and perform further debridement is advisable in equivocal cases. Options for wound closure include delayed primary closure, negative-pressure wound therapy, dermatotraction, or split-thickness skin grafting, depending on swelling and soft-tissue conditions. Renal-protective measures should be instituted early when rhabdomyolysis is present [11].

We identified 26 previously reported cases of WLCS after orthopedic or trauma surgery in the English-language literature (Table 1). The number of published cases has increased in recent decades, which may indicate that this complication remains under-recognized and under-reported.

**Table 1:** Previously reported WLCS cases in orthopedic or trauma surgery.

Study	Age	Sex	BMI	Diagnosis	Index procedure(s)	Position/support (well leg)	Surgery time	Time to diagnosis	Fasciotomy approach	Outcome/recovery
Dugdale *et al.* [12] Case 1	20	M	Not specified	Comminuted femoral fracture, left	Femoral intramedullary nailing	Hip and knee flexion of 90°, hip abduction. Calf suspended in a broad well-padded sling.	5 h 45	Not known	Two‑incision, four compartments	Moderate weakness in anterior compartment, mild contractures in deep posterior compartment at 5 months
Case 2	23	M	Not specified	Comminuted femur and neck fracture, left	Femoral intramedullary nailing	Flexion, abduction, external rotation of the right hip. Calf in a well-padded stirrup splint	6 h	Not known	Two-incisions, four compartments	Mild sensory and motor deficits in leg and foot, resolving at 6 months
Anglen *et al.* [13] Case 1	21	M	Not specified	Comminuted proximal femoral shaft fracture, right	Intramedullary nailing, distal screws breakage. Exchange nailing. Injury of the femoral artery	Hip flexion and abduction, knee flexion, leg padded with towels on a holder	6 h	18–24h later, suspicion of deep vein thrombosis	Two-incisions, four compartments	Well-healed skin graft on lateral incision. Ankle stiffness. Normal sensation on the sole of the foot at 2 years
Case 2	28	M	Not specified	Femoral shaft fracture, open tibia fracture Gustilo II, side unknown	Femoral nailing, tibial debridement and external fixation	Hip flexion and abduction. Knee flexion. Leg held by a well-padded stirrup	6 h 15	The next day	Two-incisions, four compartments	Well-healed skin grafts. Complete loss of active ankle dorsiflexion, anesthesia on the dorsum of the foot. Sticks and orthosis at 4 months
Carlson *et al.*[14] Case 1	17	M	Not specified	Fracture of the femurs (not more)	Femoral intramedullary nailing, both sides	Left first. Hip flexion 90°, abduction 40°, external rotation 40°. Knee flexion 90°. Leg holder	Less than 3 h 30	16 h	Not specified	Recovery of 4/5 strength several months after
Case 2	18	M	Not specified	Fracture of the femurs (not more)	Femoral intramedullary nailing both sides	Similar	Similar	20 h	Not specified	Peroneal function 100% loss, 100% recovered
Adler *et al.*[15] Case 1	19	M	Not specified	Femoral shaft fracture, left	Femoral intramedullary nailing 36h after trauma	Hemi-lithotomy position. Leg in a well-padded leg holder.	5 h 30	Immediately post-operatively: pain, abnormal intracompartmental pressures	Two‑incisions, four compartments	Unknown. Three debridements before closure
Case 2	29	M	Not specified	Subtrochanteric hip fracture on a past femoral nailing, right	Nail removal, open reduction and internal fixation with reconstruction nail	Hemi-lithotomy Leg on well-padded leg holder.	4 h 30	1 hpost-operatively: pain and abnormal intracompartmental pressures	Two‑incisions, four compartments	Chronic burning pain in his left forefoot
Case 3	37	M	Not specified	Non-union of femoral shaft fracture, right	Intramedullary nailing, debridement of non-union, bone graft	Hemi-lithotomy position. Leg in a well-padded leg holder.	7 h	1 h post-operatively: pain and abnormal intracompart-mental pressures	Two‑incisions, four compartments	Five debridements, skin graft before closure
Mathews *et al.* [16] Case 1	30	F	36.4 kg/m^2^	Open proximal femur fracture, right	Nail, nail exchange, nail exchange with plate and Ilizarov.	Hip flexion 80°, abduction 20°, knee flexion 70°. Leg in a sling oFlex bias stockinette, suspension with Flexoam (pad inserts) left leg height 20–25 cm above heart level	7 h 30	Extubating. Pain and abnormal intracompartmental pressures	Two‑incision, four compartments	Skin graft, posterior compartment contractures, ankle motion in flexion/extension 10-0-30°, diminished sensation on plantar side of left foot at 8 months
Case 2	18	F	34.5 kg/m^2^	Comminuted femoral shaft fracture and left sacro-iliac dislocation	Femoral intramedullary nailing	Hip flexion 90°, abduction 30°, knee flexion 80°. Leg in similar sling. Right leg height 25–30 cm. heart level.	6 h, 4 h of leg rise	6 h post-operatively	Four compartments. Revision fasciotomy 12h later	Foot dorsiflexion and hallux extension M3, decreased sensation on dorsum of foot at 14 months
Christodoulou *et al.* [17] Case 1	21	M	Not specified	Gustilo II pertrochanteric and shaft femur fracture, right	Femoral intramedullary nailing	Hemi-lithotomy position. Leg in Allen Stirrup supposed.	Less than 5 h	12 h post-operatively. Hypoesthesia, abnormal compartment pressures	Two-incisions, four compartments	Recovery of hypoesthesia
Case 2	44	M	Not specified	Subtrochanteric femoral fracture + trimalleolar ankle fracture	Femoral intramedullary nailing	Hemi-lithotomy position. Leg in Allen Stirrup supposed.	3 h	3 h post-operatively	Two‑incisions, four compartments	Partial loss of hallux dorsiflexion and edema at 1 year
Meldrum *et al.* [18] Case 1	22	M	Not specified	Comminuted subtrochanteric femur + humerus and radius	Femoral intramedullary nailing	Leg in a well-padded stirrup	3 h 15 with total anesthesia time of 10 h	In recovery room: pain and abnormal compartment pressures	Two‑incisions, four compartments	Complete recovery at 3 years
Case 2	23	M	Not specified	Comminuted subtrochanteric	Femoral intramedullary nailing	Leg in a padded stirrup	3 h 45	Before extubating: abnormal compartment pressures	Not specified	Shortening 2 cm, paraesthesia. Pain when standing for 3 h or more at 2 years
Weber *et al.* [19]	49	F	44.8 kg/m^2^	Comminuted subtrochanteric and neck fracture	Femoral intramedullary nailing	Hemi-lithotomy	2 h 55	18 h later. Pain and abnormal compartment pressures	Not specified	Loss of sensitivity in peroneal superficial nerve area, weakness in foot pronation
Noordin *et al.* [20]	35	M	28.8 kg/m^2^	Midshaft femoral fracture	Intramedullary femoral nailing within 20 h after trauma	Hip flexion 90°, 40° abduction, 40° external rotation. Knee flexion 90°. Leg in a well-padded calf rest (Lloyd-Davis leg holder) ankle free. Left leg height 45 cm. heart level	4 h 15	14 h post-operatively: hypoesthesia in peroneal area and tense anterior compartment	Two-incisions, four compartments	Recovery in 24 h, no mechanic or neurological sequelae at 7 months
Singisetti [21]	25	F	Not specified	Open tibia fracture left and splenic laceration	Tibial nailing and explorative laparotomy with splenic laceration repair	Hemi-lithotomy, leg holder with compression stocking	5h	Not specified	Not specified	No significant sequelae
Meena *et al.* [22]	28	M	37 kg/m^2^	Subtrochanteric (AO 33-C1)	Intramedullary nailing, then dynamic condylar screw within 48 h after	Leg flexion 70°, abduction 40°, leg placed in a leg holder.	2.5 h	4 h	Two‑incisions, four compartments	Union at 12 weeks; returned to work at 1 year
Hsu *et al.* [23]	28	F	20.2 kg/m^2^	Comminuted femoral shaft fracture, right	Closed reduction and stabilization with intramedullary nail	Supine hemi-lithotomy position. Leg was held by a boot and positioned in 80° of hip flexion, 30° of abduction, and 105° of knee flexion without any leg holders or fixation straps around the knee	4 h	Not specified	Conservative treatment	Complete recovery of the non-operated leg without permanent sequelae was ob- served at the 3-month follow-up
Clarke *et al.* [24] Case 1	53		Not specified	Peritrochanteric fracture of the right femur with ipsilateral femoral and tibial shaft fractures	Interlocking tibia nailing. Retrograde femoral nail	Hemi-lithotomy position with the hip flexed, abducted, externally rotated and the knee flexed at 90°	6 h 15	During the transfer post-operatively	Two‑incisions, four compartments	8 months almost full recovery with M5- power in all muscle groups and normal sensation
Case 2	17	M	21.2 kg/m^2^	Polytraumatised patient, femoral fracture (AO 32-C1), right	Diagnostic laparoscopy and femoral intramedullary nailing	Hemi-lithotomy	4 h 49	1 h post-operatively	Two-incisions, four compartments	Complete recovery at 4 months
Shultz *et al.* [25]	42	F	Not specified	Comminuted subcapital femoral neck fracture, right	Femoral neck ORIF (dynamic hip screw and cannulated screw)	Left leg in a well-padded well leg holder, positioning the knee and hip at 45° of flexion. Sequential compression device on the non injured leg.	1 h 49	In the recovery room	Selective anterior and lateral fasciotomies	Full recovery at 6 months
Anwer *et al.* [1]	18	M	Not specified	Femoral midshaft fracture, left	ORIF	Lithotomy	2 h 30	Early	Two-incisions, four compartments	Right foot motor function and sensation improvement
Ntontis *et al.* [4] Case 1	19	M	Not specified	Floating knee, left	Retrograde intramedullary nailing of the femur and an antegrade intramedullary nailing of the tibia	Lithotomy, right leg applied in 100° of the hip flexion and 90° of the knee flexion	4 h 20	3 h	Not specified	Extensor hallucis longus motor deficit M3/5, no sensory deficit at 6 months
Case 2	62	M	Not specified	Medial degenerative arthritis, left	Medial unicompartmental knee prosthesis	Lithotomy	1 h 30	3rd postoperative day - right buttock compartment syndrome	Conservative treatment	No deficits at 6 weeks

Our three cases shared the principal risk combination described in the literature: hemi-lithotomy positioning and continuous elevation with calf support of the well-leg exceeding 4 h. In one case, marked intraoperative hypotension was also present. These observations are consistent with current understanding of the pathophysiology of WLCS.

To our knowledge, the young patient of 15 years old represents the youngest individual with reported WLCS occurring in the hemi-lithotomy position in the context of orthopedic trauma described in the literature, thereby extending the current understanding of the age limits at which WLCS can develop. Particular vigilance is required in children and adolescents, as compartment syndrome can be more difficult to recognize; instead of clearly localizing pain, children may predominantly exhibit non-specific signs such as anxiety, agitation, or behavioral changes.

Although orthopedic trauma procedures may legitimately require operative times of 4 h or longer, current recommendations advise lowering and mobilizing the non-operative limb at least every 2 h to mitigate the rise in compartment pressures – an intraoperative measure that was not performed in our cases.

Diagnosis remains primarily clinical. Once WLCS is suspected, surgical decompression should not be delayed. A two-incision, four-compartment fasciotomy is recommended to avoid missed compartment involvement. Although wound morbidity is common, it is generally manageable with negative-pressure wound therapy and, when required, split-thickness skin grafting. In severe cases, prompt management of rhabdomyolysis is essential to protect renal function. Prevention measures are pragmatic and include limiting uninterrupted hemi-lithotomy time, preferring heel or ankle support, maintaining the foot in neutral positioning or slight plantarflexion, avoiding Trendelenburg positioning, preserving adequate diastolic pressure and intravascular volume, and training operating and recovery room teams to treat any new pain in the non-operative leg as WLCS until proven otherwise.

WLCS is a rare, but potentially devastating complication of orthopedic and trauma surgery. It appears to be largely preventable. Improved awareness of established risk factors among the treating personnel can enhance early identification and may reduce the incidence of preventable cases. Diagnosis is mainly clinical, and once the diagnosis is made, fasciotomy must not be delayed.
